# Laboratory Diagnosis and In Vitro Antifungal Susceptibility of *Trichophyton quinckeanum* from Human Zoonoses and Cats

**DOI:** 10.3390/antibiotics11060739

**Published:** 2022-05-30

**Authors:** Dominik Łagowski, Sebastian Gnat, Mariusz Dyląg, Aneta Nowakiewicz

**Affiliations:** 1 Department of Veterinary Microbiology, Faculty of Veterinary Medicine, University of Life Sciences, 20-033 Lublin, Poland; aneta.nowakiewicz@up.lublin.pl; 2 Department of Mycology and Genetics, Faculty of Biological Sciences, University of Wroclaw, 50-137 Wroclaw, Poland; mariusz.dylag@uwr.edu.pl

**Keywords:** cats, *Trichophyton quinckeanum*, zoonoses, asymptomatic pets, antifungal susceptibility, diagnostics

## Abstract

The “One Health” concept increasingly demonstrates the global spread of pathogenic (also eukaryotic) microorganisms and their zoonotic potential. Dermatophytes can cause superficial mycoses in humans and animals. Furthermore, the number of transmissions from asymptomatic carriers to humans has been on the rise over the last few years. This study was focused on the detailed characterisation of clinical isolates of *Trichophyton quinckeanum* with epidemiological analyses and characterisation of their in vitro antifungal susceptibility patterns. The isolated dermatophytes were identified with a combination of conventional and molecular methods. In turn, their susceptibility in vitro was tested according to the Clinical and Laboratory Standards Institute (CLSI) M38 ed.3 protocol. A total of 36 strains were isolated, with 21 cases of *T. quinckeanum* zoonoses resulting from direct contact with symptomatic cats (58.3%). The other 15 strains (41.7%) were isolated simultaneously from healthy cats and their owners. All strains showed high susceptibility to allylamine, pyridinone, and phenyl morpholine derivatives but were resistant to fluconazole and ketoconazole. In conclusion, our study shows the frequency of zoonoses contracted from asymptomatic cats. Moreover, the antifungal susceptibility profiles indicate the serious risk posed to animal owners by resistant strains of *T. quinckeanum*, which are often responsible for recalcitrant-to-treatment cases.

## 1. Introduction

Over the last few years, dermatophytoses have been transmitted from animals to humans, and there has been a concomitant increase in the number of recalcitrant-to-treatment cases worldwide, posing as a serious public health problem [1]. It is well-known that even 50% of human superficial infections might be caused by zoophilic dermatophytes, and more than half of these cases are associated with pets [2,3]. Among the pet animals, cats and dogs, in particular, are perhaps the most frequently related with the symptomatic dermatophytosis, but more often, these are asymptomatic carriers of zoophilic dermatophytes in urbanised cities [4]. Breeding animals in an apartment in a block of flats are resulting in a much close relationship between owners and pets and, consequently, a higher exposure to infection agents [1,5]. Moreover, therapy of dermatophytoses imposes a high economic burden, as approximately USD 1.67 billion is spent on antifungals each year [6].

In traditional terms, three ecological groups of dermatophytes have been described so far: anthropophilic, zoophilic, and geophilic [7]. Zoophilic species naturally colonise animals, and their transfer to humans is not only possible but quite frequent. It usually occurs through reservoirs, which may be the animals themselves, their fur hairs, or objects with which they came into contact [8]. Zoophilic dermatophytes isolated from animals may be responsible for both symptomatic infections as well for asymptomatic colonisation, making the animals asymptomatic carriers which may become a source of the epidemic [9]. However, the reservoirs of dermatophytes can change, well-established hosts for the various species are not permanent, and some of these pathogens also inhabit the soil as a favourable environment [10,11,12].

The zoophilic dermatophyte *Trichophyton quinckeanum*, earlier known as a *T. mentagrophytes* var. *quinckeanum,* was so far most often isolated in the Middle East from rodents and camels considered the main natural reservoir of this species [13]. In Europe, this pathogen has been only sporadically noted as an etiological factor of human dermatophytosis [14]. Although many controversies arise on the subject of the taxonomic status of *T. quinckeanum*, currently, molecular biology criteria clearly indicate that this pathogen constitutes a distinct species closely related to anthropophilic *T. schöenleinii* [15]. Nevertheless, its reservoirs seem to be underestimated and require verification.

An increasing number of cases of human dermatophytoses with evidence of transmission from pets in recent years prompted an in-depth analysis of these infection outbreaks. Hence, the aim of this study was a detailed phenotypic and molecular identification of clinical isolates obtained from confirmed zoonoses, their epidemiological study, and characterisation of antifungal susceptibility patterns.

## 2. Results

A total of 36 *T. quinckeanum* strains were isolated from humans (21/36) and cats (15/36) (Table 1). All patients reported contact with cats in the past as owners sharing a common area with their pets. Nine patients used to allow their cats to leave homes freely, and eight used to take the animal to allotments, recreational areas, or walks. None of the patients had any personal or professional dealings with other animals. The diagnosed infections included tinea corporis (12/21; 57%), tinea capitis (8/21; 38%), and tinea faciei (1/21; 5%). These zoonoses were more frequently noted in females (13/21; 62%) than in males (8/21; 38%). The age range of patients was 23–81 years, with a median age equal to 62 years. In 16 (76%) cases, the patients lived with other family members, predominantly with adults (74%) and their children and adolescents (≤18 years of age) (26%). None of the patients’ family members had symptoms of infection. Almost all cases were detected during the spring (15/21; 71%) and summer (4/21; 19%) months. Of the total 36 strains (41.7%) of *T. quinckeanum*, 15 were isolated simultaneously from asymptomatic cats and their owners.

**Figure 1:**
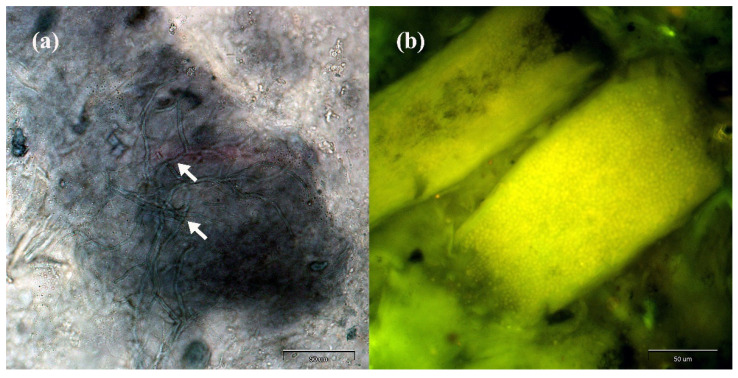
Direct preparation from human skin scrapings and hairs stained with chlorazol black E (**a**) and calcofluor white (**b**) magnified 400× (Olympus BX51, Tokyo, Japan): (**a**) mycelium fragments indicated by arrows; (**b**) arthrospores indicated by green fluorescence.

Direct analysis of the material sampled from the clinical lesions revealed the presence of filamentous fungi hyphae in the samples collected from humans and animals with skin lesions as well as asymptomatic carriers (Figure 1). 

**Figure 2:**
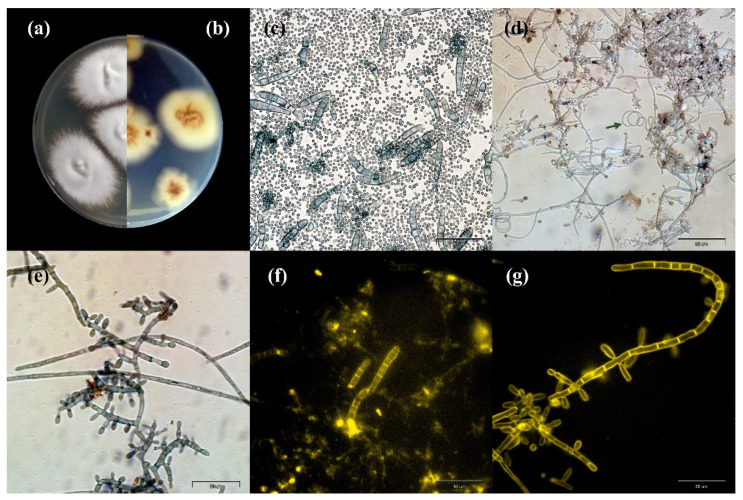
Macro- and micromorphology of *Trichophyton quinckeanum* isolated from humans and cats: (**a**,**b**) macromorphology on Sabouraud medium at 28 °C for 14 days: (**a**) obverse; (**b**) reverse; (**c**–**g**) macro- and microconidia in a 14-day culture preparation: (**c**) macro- and microconidia stained with chlorazol black E, magnification 400×; (**d**) microconidia, the arrow indicates the presence of a spiral hypha, chlorazol black staining, magnification 400×; (**e**) microconidia, chlorazol black staining, magnification 1000×; (**f**) macroconidia, calcofluor white fluorescent staining, magnification 400×; (**g**) microconidia, calcofluor white fluorescent staining, magnification 1000×.

**Figure 3:**
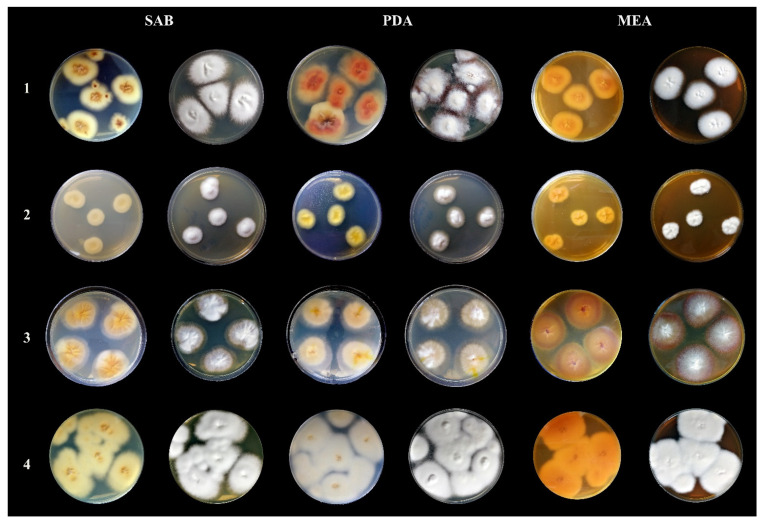
Macromorphological characteristics of *Trichophyton quinckeanum* compared with other species of dermatophytes classified under the genus *Trichophyton*. All photos show colonies from a 14-day culture at 25 °C. Mycological media: SAB—Sabouraud glucose agar, PDA—potato dextrose agar, MEA—malt extract agar. Designations of species of dermatophytes: **1**—*Trichophyton quinckeanum*, **2**—*Trichophyton schöenleinii*, **3**—*Trichophyton benhamiae*, **4**—*Trichophyton mentagrophytes*.

The observed macro- and micromorphological features were characteristic of *T. quinckeanum* (Figure 2 and Figure 3). The colony diameter ranged from 32 to 44 mm at 25 °C and 37 °C, respectively, after 10 days. Longer incubation did not increase the size of the colony. The colony colour was white to dark-greyish purple, and the reverse was vivid orange to yellowish brown. The colonies characteristically spread rapidly; they were flat or slightly elevated in the centres. The colony edges were star-shaped, and their texture was granular to velvety. The microscopic observations revealed the presence of numerous macroconidia, which dominated the other fungal elements. The macroconidia were thin-walled, cigar-shaped, or clavate. They formed at the end of hyphae and consisted of 6–8 chambers. The microconidia were predominantly pyriform to clavate. In older cultures (≥14 days of incubation), spiral hyphae were also visible in the microscopic preparations.

The nucleotide sequences of the ITS rDNA region were identical in all the examined *T. quinckeanum* strains, regardless of whether they were obtained from humans or animals. The representative sequence was deposited in the GenBank database under accession number MZ695772 (strain TQ1 obtained from patient no. 1). The nucleotide sequences obtained from clinical isolates showed 100% similarity to reference strain *T. quinckeanum* IHEM26522 (MK298974). However, the ITS sequences of *T. quinckeanum* isolates revealed only two substitutions and 99.51% similarity in comparison to the reference strain of anthropophilic *T. schöenleinii* CBS564.94 (MN808784). 

The results of the antifungal susceptibility testing of *T. quinckeanum* clinical isolates obtained from humans and animals are given in Table 2. Allylamine exhibited the lowest MIC_50_ and MIC_90_ values in comparison with the other tested antifungals, regardless of the dermatophyte host. In turn, fluconazole was found to exert the weakest in vitro effect and had the highest MIC_50_ and MIC_90_ values. Additionally, in the case of all the *T. quinckeanum* strains isolated from humans, fluconazole had the widest while naftifine and terbinafine had the narrowest MIC range, i.e., 2–32 μg/mL and 0.004–0.016 μg/mL, respectively. The MIC_90_ of amorolfine, clotrimazole, ciclopirox, enilconazole, itraconazole, miconazole, naftifine, terbinafine, and voriconazole in the case of all *T. quinckeanum* isolates were below 1 μg/mL, whereas those of ketoconazole as well as MIC_50_ and MIC_90_ of fluconazole were above 1 μg/mL.

## 3. Discussion

In Europe, an increase in the incidence of zoophilic dermatophytoses has been noted in recent years despite improvement in living standards, greater attention to hygiene, and the development of medicine [16,17]. Commonly, superficial infections caused by dermatophytes are mostly seen in those with low socioeconomic status, practicing certain sports, agricultural, or veterinary professions, and are also associated with climatic factors [18]. Furthermore, *T. quinckeanum* has been only sporadically diagnosed in samples collected from humans. The incidence of mycosis related to this pathogen was described in the literature in 2018 by Uhrlaß et al. [13] in Germany. Moreover, in Czechia, Lysková et al. [19] described two human cases of *T. quinckeanum* dermatophytosis in 2017, four in 2018, eight in 2019, and ten in 2020. No similar cases have been reported so far in other European countries, including Poland. 

Nevertheless, the occurrence of this dermatophyte in Poland may not be a new occurrence in terms of recent decades in contrast to the absence of *T. quinckeanum* in previous years. It may rather be the result of routine identification of dermatophytes based mainly on ITS rDNA region sequencing [15]. For this reason, differentiation of closely related species cannot be carried out only by analyses of nucleotide sequences of ITS rDNA regions. Laboratory diagnostics should also refer to the different morphology and ecology of *T. quinckeanum* and *T. schöenleinii*. Our study confirms previous observations that this molecular marker is not suitable to differentiate zoophilic *T. quinckeanum* from anthropophilic *T. schöenleinii* [20,21]. However, from the taxonomic point of view, these two species have always been regarded as different entities, and in fact, *T. quinckeanum* and *T. schöenleinii* are two distinct pathogenic fungi, which probably share a common saprophytic ancestor with the geophilic species *Arthroderma simii* [21]. The differences between these two species are clearly visible at the morphological level. *Trichophyton quinckeanum* produces numerous microconidia and macroconidia, whereas *T. schöenleinii* rarely or even never produces these structures in standard cultivation conditions in a mycological laboratory, i.e., at 25 °C on Sabouraud dextrose agar (SDA), potato dextrose agar (PDA), or malt extract agar (MEA) after 14 days of incubation [22]. In this aspect, it should be noted that the analysis of the morphology of the isolates in our study cannot completely rule out the possibility that this pathogen was misidentified due to its morphological similarity to *T. mentagrophytes* [23]. Uhrlaß et al. [13] showed that the matrix-assisted laser desorption and ionisation mass spectrometry (MALDI–TOF MS) technique, in combination with conventional diagnostic procedures, supports the possibility of differentiation of *T. quinckeanum* from other common zoophilic species, such as the *T. mentagrophytes* and *T. benhamiae* complexes of species. Nevertheless, the application of this method routinely requires further detailed studies due to some uncertainties in the obtained results [19]. Packeu et al. [24] revealed that one out of six *T. quinckeanum* clinical isolates was incorrectly identified as *T. schöenleinii* using MALDI–TOF MS. Other diagnostic features that may be taken into account are clinical lesions caused by the pathogens. *T. schöenleinii* is principally related to favus on the scalp, whereas symptoms produced by *T. quinckeanum* in humans are mainly localised on glabrous skin. Nevertheless, both of them typically manifest themselves by scutula formation [13]. Fortunately, in contrast to *T. schöenleinii*, *T. quinckeanum* infected human hairs do not fluoresce under Wood’s ultra-violet light. Hence, discrimination between these two species is not a simple task, even for experienced mycologists.

The emergence of *T. quinckeanum* infection cases in humans in recent years is in concordance with the fashion for breeding pets at home. Therefore, it seems that asking a question in the medical history regarding contact with animals is essential information to make a proper diagnosis. In general, *T. quinckeanum* is historically connected with mouse or rodent favus [25,26]. Currently, cats, dogs, rabbits, camels, chickens, horses, and sheep are the occasional hosts for this dermatophyte as well [13,19]. This is understandable in the case of cats, as they often come into direct contact with mice and rats. Our study has shown that cats can be an underestimated source of zoonoses with *T. quinckeanum* as the etiological factor. In their case report from Germany, Uhrlaß et al. [13] also observed that cats were the main source of infection which is in agreement with our data. These observations are confirmed by Lysková et al. [19] on the basis of research carried out in 2017–2020. Moreover, in addition to cats, these authors isolated *T. quinckeanum* from dogs. It is interesting that, in our study, all the 15 asymptomatic cats showed positive mycological test results, including obtaining a dermatophyte culture. The carrying of dermatophytes is common among pets, especially cats, and poses a significant risk of mycosis outbreaks [16,27]. *Trichophyton quinckeanum* should also be indicated as a potential species that may occur in the carrier status of cats and dogs and exhibit a high zoonotic risk.

Dermatophytoses probably easily spread among animals, as indicated by the occurrence of infection in several animals in the same household [28,29]. The spread of infections among domestic animals is probably the main driving force of the current outbreak in other countries, which is similar to the German one [13]. However, in our study, the sudden rise of *T. quinckeanum* infections may not be easily explained. The patients had close contact with only one animal, i.e., cats, and transmission of the infection to other domestic animals was excluded. Nevertheless, some of the animals may have left their homes freely or have left the house with the owners. Therefore, the primary source of infections can be searched in the overpopulation of rodents in urban areas, potentially leading to transmission to domestic animals and further to humans [30]. One might also consider soil as a possible reservoir of this dermatophyte. Some zoophilic dermatophytes, such as *Nannizzia nana*, change their ecological niches and are commonly found in soil, where pathogens can be mechanically transferred by pets to their owners [12,31]. However, more detailed comparative studies are needed for such conclusions to be drawn.

In our study, we found that the majority of patients were elderly people, and the median age was 62 years. This observation is in line with previous results. Uhrlaß et al. [13] recorded that the majority of patients were older than 50 years. Moreover, in agreement with Uhrlaß et al. [13], our study confirms the higher infection rates of this zoonosis in females. It seems that both factors, i.e., older age and sex can be explained by the fact that women more often are in close contact and perform hygienic activities while taking care of their pets. In turn, in the Czech patients suffering from *T. quinckeanum* dermatophytosis, Lysková et al. [19] revealed infections mostly in the age groups of 19-to-49 years (48%) and 1-to-18 years (44%), with only two patients being older than 50 years. These discrepancies may be due to different lifestyles and cultural attitudes towards companion animals in European countries.

Dermatophytoses caused by *T. quinckeanum* in humans are usually treated with a combination of various oral and topical antifungals. According to the literature, griseofulvin, terbinafine, clotrimazole, ciclopirox, and ketoconazole were efficiently used in the therapy [14,32,33]. Skorepová et al. [34] reported failure in the treatment of this mycosis with bifonazole creams. The patients in our study were successfully treated with terbinafine, clotrimazole, ciclopirox, and fusidic acid used as part of 21-day therapy based on an oral and topical combination of these antifungals. The antifungal susceptibility tests with an endpoint value of 80% showed that clinical isolates of *T. quinckeanum* have a high susceptibility to allylamine, pyridinone, and phenyl morpholine derivatives. Among the 36 tested clinical isolates of *T. quinckeanum*, 28 and 7 strains demonstrated resistance towards fluconazole and ketoconazole, with MIC values ≥8 µg/mL and ≥1 µg/mL, respectively (Table 2). In the literature, there are only fragmentary results of antifungal susceptibility tests in relation to *T. quinckeanum*. Niewerth et al. [35] tested one strain of *T. quinckeanum* for itraconazole, terbinafine, and ciclopirox and reported high susceptibility, with MIC values equal to 0.001 µg/mL, 0.01 µg/mL and 0.03 µg/mL, respectively. In turn, symptomatic animals were successfully treated with miconazole or terbinafine without or in combination with chlorhexidine, enilconazole, and sulphur lyme by applying whole-body baths three times a week for 14 days. Moreover, the MIC values for isolates obtained from samples taken from cats were similar to those obtained for human isolates.

A limitation of this study can be the fact that it consisted of an analysis of only cats as companion animals, which are very commonly kept in small-sized apartments. However, it should be noted that in Poland in 2020, the average size of a flat in a multifamily block was 52.8 m^2^, with on average 2.34 people living together, as shown by the data of the Central Statistical Office (GUS). The space of the apartments is, therefore, small and contact with animals, due to their nature, is probably high. It should be taken into account that 37% of Polish residents share their house or apartment with a cat, and 43% of all owners keep two or more cats [36]. The epidemiological situation is aggravated by the fact that a total of 66% of city cats are allowed to leave their homes freely, and 11% spend the vast majority of their time outside the home. In addition, 35% of cats in Poland are young cats, which are especially predisposed to infections or carriage of dermatophytes. These data support the necessity of monitoring studies. Nevertheless, as demonstrated by Lysková et al. [22], on the basis of their studies carried out in the Czech Republic, *T. quinckeanum* was also isolated from dogs. The host range of this species may be much wider, and analysis should not be limited to cats. However, the presented results indicate the need to consider zoonosis in each case of dermatophytosis and keep cats or other companion animals inside apartments in blocks of flats.

## 4. Materials and Methods

### 4.1. Study Design

The present study is a case–control study. The collection of the material was based on the analysis of cases of dermatophytosis in humans with a history of contact with a cat bred in an apartment in a multifamily block of flats. In the next stage, clinical signs of dermatophytosis in cats were analysed. The material was collected both from cats with visible symptoms of dermatophytosis and from cats without symptoms, which, in the case of a positive result, were referred to as carriers.

### 4.2. Dermatophyte Clinical Isolates

In total, 21 clinical isolates of *Trichophyton quinckeanum* were isolated as etiological agents of human dermatophytoses across Poland in 2018 (Table 1). All of these cases were classified as zoonoses and related to patients contacted with symptomatic cats living with their owners in flats in different cities. In 15 cases, the dermatophytes were also isolated from the asymptomatic cats after infection was confirmed in their owners. Within this group, there were no symptomatic cats living with these patients. In total, 36 clinical isolates of *T. quinckeanum* were examined. Human clinical material was collected especially from the margins of skin lesions using a sterile surgical scalpel. In the case of cats, sampling was performed using the brush technique.

### 4.3. Diagnosis Procedure

#### 4.3.1. Direct Microscopical Examination

A direct examination of collected hairs, skin and nail scrapings, and phenotypic characteristics was performed based on comparative analyses of macro- and micromorphology of cultivated fungi and molecular biology methods. The last-mentioned factor allowing for unique molecular identification to the species level was based on sequencing of the PCR product obtained with ITS1 and ITS4 pair of primers complementary to the rDNA gene cluster. For direct microscopical examination of the clinical material collected from the patients’ clearing fluid, a solution comprising dimethyl sulphoxide (DMSO) and 10% KOH was used. For better visualisation, additional staining with lactophenol blue (Sigma-Aldrich, Saint Louis, MO, USA) or calcofluor white (Sigma-Aldrich, Saint Louis, MO, USA) was performed. The preparations were examined in the presence of any fungal elements under a light or fluorescence microscope (Olympus BX51, Tokyo, Japan). In each microscopic preparation, 10 visual fields were examined under a magnification of 400×.

#### 4.3.2. Primary Isolation on Culture

For the primary isolation of dermatophytes from clinical samples, Sabouraud glucose agar (SAB; BioMaxima, Lublin, Poland) was used, supplemented with 0.05% chloramphenicol, 0.04% gentamicin, and 0.5% cycloheximide. Pure isolates of *T. quinckeanum* were cultured on SAB, potato dextrose agar (PDA; Oxoid, Basingstoke, UK), and malt extract agar (MEA; BioMaxima, Lublin, Poland) at 25 °C and 37 °C for 21 days and were analysed macro- and microscopically every 3 days. The fungi were identified based on colony texture, presence of typical mycelial structures, and species-specific macroconidia, according to de Hoog et al. [23].

#### 4.3.3. Molecular Identification 

Genomic DNA was extracted, as described previously by Gnat et al. [37]. The quality of the extracted DNA was evaluated with a NanoDrop 1000 spectrophotometer (Thermo Scientific, Waltham, MA, USA). Control extractions were performed using nuclease-free water (Thermo Scientific, Waltham, MA, USA) instead of cell suspension based on the same protocols. The ITS rDNA region (ITS1-5.8S-ITS2) was amplified using the ITS1 and ITS4 primer pairs [38] (ITS1: 5′-TCCGTAGGTGAACCTGCGG-3′ and ITS4: 5′-TCCTCCGCTTATTGATATGC-3′). PCR reaction for ITS rDNA region amplification was carried out using a T Personal thermal cycler (Biometra GmbH, Göttingen, Germany), with 25 µL of total volume reaction mixture composed of 12.5 µL QIAGEN Taq PCR Master Mix (2.5 U Taq DNA Polymerase, 200 pmol of each nucleotide, and 1.5 mmol MgCl_2_) (QIAGEN, Hilden, Germany), 10 pmol of each primer (Genomed S.A, Warsaw, Poland), and 1 µL of DNA template. The PCR reaction conditions were as follows: initial denaturation cycle at 95 °C for 3 min followed by 30 cycles comprising proper denaturation at 95 °C for 1 min, annealing at 50 °C for 1 min, and elongation at 72 °C for 1 min, followed by final extension cycle at 72 °C for 10 min and termination at 4 °C. Electrophoretic separation of PCR products was carried out in 1% agarose gels. The PCR products sequencing reaction was carried out using a BigDye Terminator Cycle Sequencing Kit (Life Technologies, Carlsbad, CA, USA) with mentioned primers ITS1/ITS4 each time used solely. The PCR mixture (10 µL) contained 2 µL of 2.5× concentrated Ready Reaction Premix, 1 µL of 5× concentrated BigDye Sequencing Buffer, 0.25 µL of one of two mentioned primers at a concentration of 5 pmol (initially 100 pmol), a DNA amplicon at a concentration of 50 ng, and nuclease-free water at a final volume of 10 µL. Two separate reactions were carried out for each one of the pair of primers ITS1/ITS4. PCR reactions were performed in a T Personal cycler (Biometra GmbH) with the following conditions: initial denaturation for 1 min at 96 °C, denaturation for 10 s at 96 °C, annealing of primers for 5 s at 50 °C, and elongation for 4 min at 60 °C. The last-mentioned three stages, i.e., denaturation, annealing of primers, and elongation, were repeated 25 times. The PCR product was purified using an ExTerminator Kit (A&A Biotechnology, Gdynia, Poland), and then the DNA sequence was read in a 3500 Genetic Analyser (Life Technologies, Carlsbad, CA, USA).

### 4.4. Antifungal Susceptibility Testing

In vitro testing of the susceptibility to allylamine, polyene, imidazole, triazole, and pyridinone derivatives, as well as phenyl morpholine derivatives, was performed according to the Clinical and Laboratory Standards Institute (CLSI) M38 ed.3 protocol [39]. Reagent-grade amorolfine (AMR), ciclopirox (CPO), clotrimazole (CLT), enilconazole (ENC), fluconazole (FLC), itraconazole (ITR), ketoconazole (KTC), miconazole (MCZ), naftifine (NFT), terbinafine (TRB), and voriconazole (VRC) were obtained in powder form (Sigma-Aldrich, MO, USA). Drug stock solutions were prepared in dimethyl sulfoxide (DMSO) and the final concentration of the last-mentioned drug did not exceed 1% in working solutions. The drugs were analysed at the final concentration in the range of 0.002–2 μg/mL for allylamine, pyridinone derivatives, and phenyl morpholine derivatives, 0.004–4 μg/mL for imidazoles, itraconazole, and voriconazole, and 0.06–64 μg/mL for fluconazole. The dermatophyte isolates were previously cultured on Sabouraud glucose agar (BioMaxima, Lublin, Poland) for 21 days, and inoculum suspensions comprising mostly conidia were prepared by gentle scraping mature colonies of dermatophytes into sterile physiological saline containing 0.002% Tween 80. Homogeneous supernatants of inoculum suspensions were collected, and their optical density (OD) at 530 nm was adjusted spectrophotometrically to 65% to 70% transmission that the final density of inoculum was in the range of 1 × 10^3^ to 3 × 10^3^ CFU/mL. Homogeneous inoculum suspensions with spectrophotometrically established density were additionally standardised based on counting in a haemocytometer (BrightLine^TM^, Sigma-Aldrich, Saint Louis, MO, USA). Inoculum suspensions prepared in this way were diluted 1:50 in RPMI 1640 medium and incubated at 35 °C for 72 h in the presence of antifungals at concentrations indicated previously, using 96-well titration plates. Minimum inhibitory concentrations (MICs) were determined spectrophotometrically considering the MIC_80_ value is the concentration of antifungal at which at least 80% growth inhibition, compared with the control (drug-free well) was observed using a SmartSpec^TM^ (BioRad, Hercules, CA, USA) at 530 nm wavelength (λ). *Trichophyton rubrum* MYA4438 and *Trichophyton interdigitale* MYA4439 reference strains served as quality controls for every new series of susceptibility tests performed according to microdilution assay. The breakpoint of MIC ≥ 1 µg/mL was used to categorise the dermatophyte strains as resistant [40]. The exception was fluconazole, for which the resistance criterion was MIC ≥ 8 µg/mL [41]. All tests were performed in triplicate, and differences between mean values were assessed by Student’s *t*-test using the R program version 3.6.3 (R Core Team, Vienna, Austria).

## 5. Conclusions

The outbreak of *T. quinckeanum* infections in humans may result from diagnostic difficulties in distinguishing this species from the anthropophilic *T. schöenleinii*. It seems, however, that whenever contact with animals is revealed, this zoophilic pathogen should be taken into account as one of the etiological factors of dermatophytoses in humans. Moreover, the rising number of human infections is mostly mediated by asymptomatic carriage including cats kept as pets. As a rule, the treatment of such cases does not cause problems, but attention should be paid to the revealed resistance to ketoconazole and fluconazole. Finally, the growing numbers of infections caused by *T. quinckeanum* underscore the need for closer collaboration between veterinarians and dermatologists to establish appropriate reservoirs and preventive measures.

## Figures and Tables

**Table 1:** Clinical isolates of *Trichophyton quinckeanum* examined in this study with description.

No. of Patients	Sex	Age	Type of Infection	Month of Isolation	Contact with Cat	Race	Leaving Home	Confirmed Infection	Treatment	
									Humans	Animals
1	F	23	tinea capitis	April	+	European Shorthair Cat	freely	asymptomatic	Terbinafine	Miconazole, chlorhexidine
2	M	62	tinea corporis	March	+	Dachshund	freely	asymptomatic	Ciclopirox, terbinafine	Enilconazole
3	F	32	tinea capitis	April	+	Siberian Cat	freely	asymptomatic	Terbinafine	Terbinafine, chlorexidine
4	F	80	tinea corporis	May	+	Ragdoll	with the owner	asymptomatic	Clotrimazole, terbinafine	Terbinafine
5	M	68	tinea capitis	October	+	European Cat	freely	asymptomatic	Fusidic acid, ciclopirox, terbinafine	Miconazole, chlorhexidine
6	F	79	tinea corporis	May	+	European Cat	freely	asymptomatic	Terbinafine, ciclopirox	Miconazole, chlorhexidine
7	F	45	tinea corporis	July	+	Dachshund	freely	asymptomatic	Fusidic acid, ciclopirox, terbinafine	Sulphur lyme
8	M	69	tinea capitis	April	+	Scottish Fold	with the owner	none	Terbinafine	NA
9	F	31	tinea corporis	June	+	Persian	with the owner	none	Clotrimazole, terbinafine	NA
10	F	70	tinea corporis	May	+	British shorthair	with the owner	asymptomatic	Terbinafine, ciclopirox, clotrimazole	Enilconazole
11	F	81	tinea capitis	March	+	European Cat	freely	asymptomatic	Fusidic acid, ciclopirox, terbinafine	Miconazole, chlorhexidine
12	F	52	tinea capitis	July	+	Archangel Blue	with the owner	asymptomatic	Clotrimazole, terbinafine	Enilconazole
13	F	74	tinea corporis	June	+	Persian	no possibility	none	Clotrimazole, ciclopirox	NA
14	F	69	tinea corporis	May	+	European Cat	freely	asymptomatic	Clotrimazole, ciclopirox	Miconazole, chlorhexidine
15	M	75	tinea faciei	March	+	Siamese	no possibility	none	Ciclopirox, terbinafine	NA
16	M	28	tinea corporis	April	+	Dachshund	freely	asymptomatic	Terbinafine, ciclopirox, clotrimazole	Miconazole, chlorhexidine
17	F	76	tinea corporis	July	+	Sphynx	no possibility	none	Clotrimazole, terbinafine	NA
18	M	60	tinea capitis	November	+	Bengal cat	no possibility	none	Clotrimazole, terbinafine	NA
19	F	32	tinea corporis	April	+	British shorthair	with the owner	asymptomatic	Terbinafine, ciclopirox	Sulfur lyme
20	M	40	tinea corporis	July	+	Abyssinian cat	with the owner	asymptomatic	Clotrimazole, terbinafine	Terbinafine, chlorohexidine
21	M	37	tinea capitis	May	+	British Longhair	with the owner	asymptomatic	Terbinafine	Enilconazole

F—female; M—male; NA—not applicable, the dermatophyte was not isolated from the animal; asymptomatic infection in cats was associated with the isolation of *Trichophyton quinckeanum* after collecting the material using the brush method; the ITS rDNA sequence with 100% similarity was obtained for each isolate from patients and animals; a sequence representative of the TQ1 isolate collected from patient no. 1 was deposited into the GenBank database, accession number MZ695772.

**Table 2:** In vitro antifungal susceptibilities of clinical isolates of *Trichophyton quinckeanum* obtained from humans and animals.

Antifungal Agents		Host	MIC (µg/mL)														MIC Range	MIC_50_	MIC_90_	MIC_GM_
			0.004	0.008	0.016	0.03	0.06	0.125	0.25	0.5	1	2	4	8	16	32				
Allylamine	NFT	humans	3	6	12												0.004–0.016	0.016	0.016	0.012
		animals		13	2												0.008–0.016	0.008	0.008	0.009
	TRB	humans	4	10	7												0.004–0.016	0.008	0.016	0.01
		animals		11	4												0.008–0.016	0.008	0.016	0.01
Imidazoles	KTC	humans						4	5	6	5						0.125–1	0.5	1	0.46
		animals						5	7	1	2						0.125–1	0.25	1	0.33
	MCZ	humans		1	5	10	5										0.008–0.06	0.03	0.06	0.03
		animals			4	11											0.016–0.03	0.03	0.03	0.03
	ENC	humans				6	12	2	1								0.03–0.25	0.06	0.125	0.07
		animals				2	8	5									0.03–0.125	0.06	0.125	0.08
	CLT	humans					3	6	8	4							0.06–0.5	0.25	0.5	0.19
		animals						1	14								0.125–0.25	0.25	0.25	0.24
Triazoles	ITC	humans				7	9	4	1								0.03–0.25	0.06	0.125	0.07
		animals					14	1									0.06–0.125	0.06	0.125	0.06
	FLC	humans										2	6	7	3	3	2–32	8	32	10.85
		animals												9	4	2	8–32	8	32	13.33
	VRC	humans				3	11	5	2								0.03–0.25	0.06	0.25	0.09
		animals				1	10	4									0.03–0.125	0.06	0.125	0.08
Pyridinone derivatives	CPO	humans					2	3	8	8							0.06–0.5	0.25	0.5	0.31
		animals						4	9	2							0.125–0.5	0.25	0.5	0.25
Phenyl morpholine derivatives	AMR	humans		6	7	7	1										0.008–0.06	0.016	0.03	0.02
		animals		1	9	5											0.008–0.03	0.016	0.03	0.02

Abbreviations of antifungal substances: AMR—amorolfine, CLT—clotrimazole, CPO—ciclopirox, ENC—enilconazole, FLC—fluconazole, ITC—itraconazole, KTC—ketoconazole, MCZ—miconazole, NFT—naftifine, TRB—terbinafine, VRC—voriconazole.

## Data Availability

All data are available from the corresponding author upon request.
